# The Dilemma of Diagnosing Hemophagocytic Lymphohistiocytosis in Sickle Cell Disease

**DOI:** 10.7759/cureus.12255

**Published:** 2020-12-24

**Authors:** Sanjay Kumar Sahu, Aman Agrawal, Palash Das

**Affiliations:** 1 Pediatrics, Kalinga Institute of Medical Sciences, Bhubaneswar, IND; 2 Pediatrics, Kalinga Institute of Medical Sciences, Bhubaneshwar, IND

**Keywords:** macrophage activation syndrome (mas), non-immune hemolytic anemia, sickle cell disease: scd

## Abstract

Hemophagocytic lymphohistiocytosis (HLH) is a life-threatening disorder due to uncontrolled activation of macrophage and cytokine release, which can be due to either genetic causes (familial) or secondary to infections malignancy and other less common cause. Parvovirus B19 rarely causes HLH. Diagnosing HLH in sickle cell disease, which inherently has high ferritin levels and pancytopenia, is particularly challenging. We are reporting HLH as a complication with parvovirus B19 infection in the background of sickle beta-thalassemia. Based on our search of available medical literature, this is the first case of HLH complicating parvovirus B19 infection in a pediatric age group with sickle beta-thalassemia.

## Introduction

Sickle beta-thalassemia is a common disorder in the eastern part of India due to the inheritance of the β globin gene mutation carrying the sickle cell deletion and thalassemia mutation [1]. These children usually present with non-transfusion dependent anemia, vaso-occlusive crisis, splenomegaly, and a higher risk of infections. Most of the patients have high ferritin levels due to the underlying pathology of anemia and ineffective erythropoiesis. They are usually treated with long-term hydroxyurea therapy. Pancytopenia is seen in some of these children due to the effect of hydroxyurea or hypersplenism.

Infection with Epstein-Barr virus (EBV) and cytomegalovirus (CMV) are usual triggers of infection-induced HLH, while parvovirus infection triggering HLH is rare [2]. We present our experience in dealing with a child with sickle beta-thalassemia presenting with features suggestive of septicemia, which got complicated by HLH but led to diagnostic difficulty due to the inherent nature of the underlying disease process.

## Case presentation

We report a case of a 12-year-old male child born out of non-consanguineous marriage admitted with the complaints of fever, vomiting, headache, chest pain, and difficulty in breathing for three days. The child was a known case of sickle beta-thalassemia from two years of age. He was diagnosed by high-pressure liquid chromatography (HPLC), which showed fetal hemoglobin (HbF) 31.6%, sickle hemoglobin (HbS) 62.4%, hemoglobin A2 (HbA2) of 3.8%. He had past history suggestive of similar complaints with hospitalizations and blood transfusions and was on treatment with hydroxyurea and folic acid for the same for the last two years. 

At the admission time, the child was febrile, pale with mild respiratory distress with normal breath sounds in both lung fields without any added sounds. There was cervical, axillary, and inguinal lymphadenopathy and enlarged liver 6 cm below the right costal margin and spleen enlarged 5 cm below the left costal margin.

Given the previous clinical diagnosis, acute chest syndrome was considered and was treated accordingly after sending relevant investigations.

Initial investigation revealed neutrophilia with normal total leukocyte count, low red blood cell (RBC) count with anemia (hemoglobin [Hb] 6.2 gm/dL), normal platelet count, Malaria parasites, and typhoid test were done and were negative. No abnormality was found in a routine urine test, an X-ray of the chest was done and was normal, and erythrocyte sedimentation rate (ESR) was 116 mm in the first hour. Serum ferritin was 3176 ng/mL, which was high, assumed due to repeated transfusions (Tables 1-2).

**Table 1:** Haematological parameters during the course of hospitalization HIV- human Immunodeficiency virus; HBsAg - hepatitis B surface immunoglobulin: HCV - hepatitis C; IgM - immunoglobulin M; ICT - immunochromatographic test

Parameter	On admission	Day 5	Day 10
Total leucocyte count (x10^3/^mm3)	9.3	4.27	5.3
Neutrophil (%)	87	20	72
Lymphocyte (%)	7	75	21
Monocyte (%)	6	3	7
Eosinophil (%)	0	2	0
Basophil (%)	0	0	0
Total red blood cell count (x10^6/^mm3)	2.41	2.34	3.41
Hemoglobin (g/dL)	6.2	6.2	8.7
Total platelet count (x10^5/^mm3)	219	80	386
Malaria parasite (ICT)	Negative		
Typhidot IgM	Negative		
HBsAg	Negative		
HIV	Negative		
HCV	Negative		
Reticulocyte (%)	0.2		
C-reactive protein (g/dL)	29.87		4

**Table 2:** Biochemical parameters during the course of hospitalization SGOT - serum glutamate oxaloacetate transferase; SGPT - serum glutamate pyruvate transferase; GGT - Gamma-glutamyl transferase; LDH - lactate dehydrogenase; ANA - antinuclear antibody; ESR - erythrocyte sedimentation rate

Biochemical parameters			
Bilirubin (total) (mg/dL)	3.03	Sodium (mmol/l)	132
Bilirubin (direct) (mg/dL)	1.4	Potassium (mmol/l)	4.8
SGOT (U/L)	98	Calcium (mg/dL)	7.7
SGPT (U/L)	43	Phosphorus (mg/dL)	6.6
GGT (U/L)	37	Uric acid (mg/dL)	6.1
Alkaline phosphate (U/L)	125	Procalcitonin (ng/ml)	4.85
Total protein (G/L)	10.5	Fibrinogen (mg/dL)	136
Albumin (G/L)	4	Ferritin (ng/ml)	3176 (2190 repeat)
LDH (U/L)	778.5	Triglyceride (mg/dL)	308
Urea (mg/dL)	36	ANA	neg
Creatinine (mg/dL)	0.4	ESR (mm in 1 hour)	116

The child was started with antibiotics (piperacillin + tazobactam with amikacin) after sending blood culture, analgesics for pain, antipyretics for fever with intravenous fluids for adequate hydration, and blood transfusion were given. After 48 hours, the child improved - vomiting, headache, chest pain subsided, and difficulty in breathing decreased, but fever persisted. Even after five days of treatment, the child continued to have a fever, so repeat investigations were sent. Blood culture and urine culture send initially were sterile.

Repeat investigations revealed worsening leucopenia, absolute neutropenia, low RBC count with severe anemia despite blood transfusion, and thrombocytopenia. Dengue immunoglobulin M (IgM) was negative. Fine needle aspiration cytology of the lymph node revealed reactive hyperplasia. IgM by enzyme-linked immunoassay and polymerase chain reaction for parvovirus B19 was positive.

**Figure 1:**
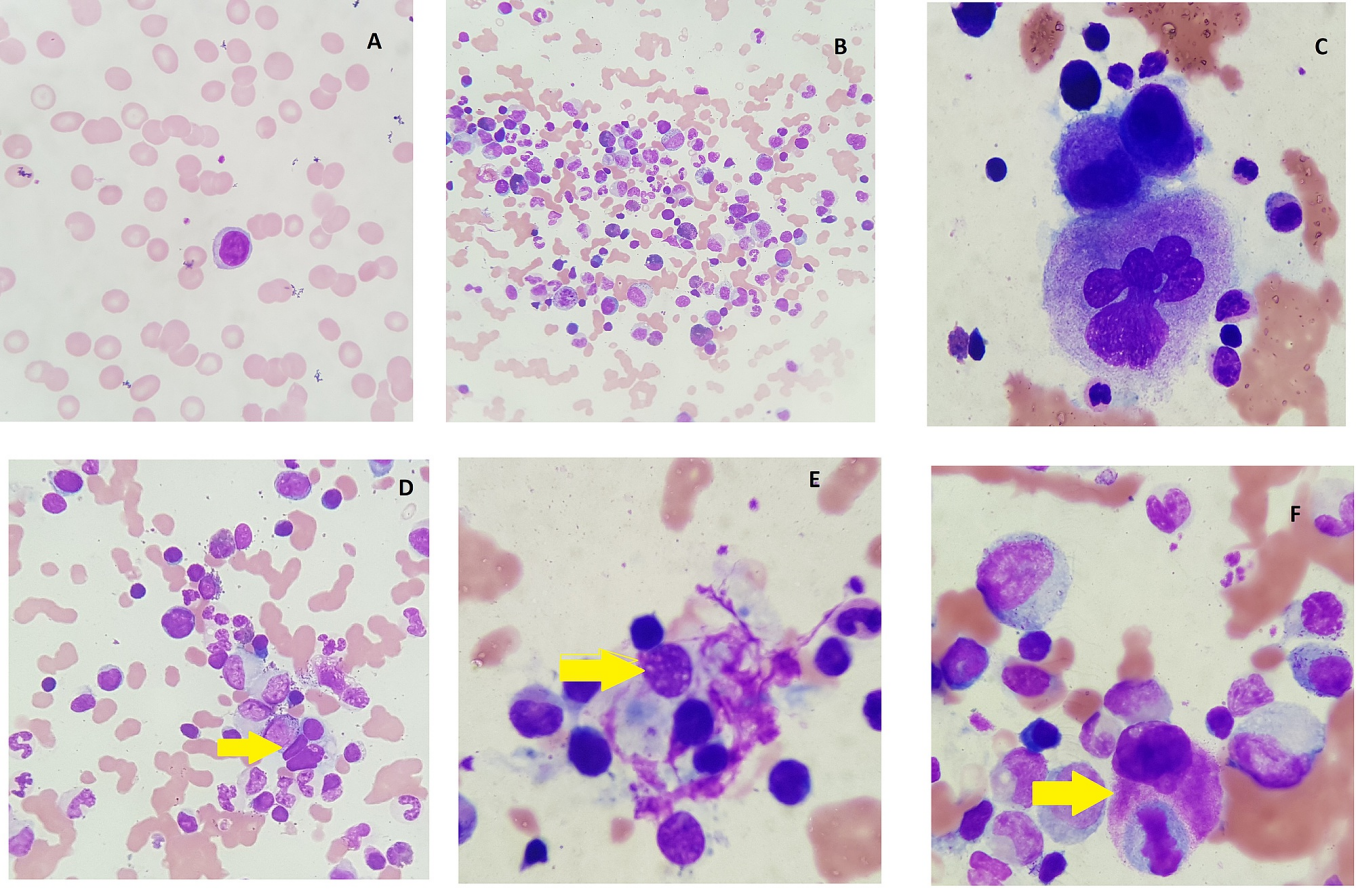
Bone marrow aspirate histopathology Pane A: Peripheral smear showing microcytic hypochromic red blood cells (RBC) with activated lymphocytes. Pane B: Bone marrow showing predominance of myeloid cells with a relative suppression of erythropoiesis. Pane C: Bone marrow aspirate showing immature as well as mature megakaryopoiesis. Pane D-F: Bone marrow aspirate showing hemophagocytosis of various hematopoietic elements by a prominent population of reticulum cells.

In view of persisting fever, pancytopenia, hyperferritinemia, and parvovirus infection, serum triglyceride and serum fibrinogen were sent. Serum triglyceride was 308 mg/dl (high), serum fibrinogen was136mg/dl (low). The bone marrow aspiration revealed haemophagocytosis (Figure 1).

The child was diagnosed as sickle beta-thalassemia with haemophagocytic lymphohistiocytosis as per the diagnostic criteria of the HLH-2004 protocol of the Histiocyte Society [3] and was started dexamethasone based on the HLH 2004 protocol. Because of pancytopenia, it was decided not to add cyclosporine or etoposide based on the progression of symptoms. The child became afebrile after 72 hours of starting dexamethasone, and after five days, anemia, leucopenia, and thrombocytopenia had an improving trend, and serum ferritin showed a decreasing trend. The child improved symptomatically and was given dexamethasone on tapering doses for eight weeks. Presently the child is symptomatically better and presently on hydroxyurea and folic acid and regular monitoring.

## Discussion

HLH is a potentially life-threatening complication if not treated. HLH is diagnosed by the HLH-2004 criteria, which demand a minimum of five out of eight criteria to be present: 1) fever >38.5°C, 2) splenomegaly, 3) cytopenia (at least two cell lineages are aﬀected), 4) hypertriglyceridemia or decreased fibrinogen, 5) ferritin >500ng/ml, 6) haemophagocytosis in bone marrow, spleen, or lymph nodes, 7) low, or absent natural killer (NK) cell activity, 8) elevated interleukin (IL) 2 receptor [3]. Hemophagocytosis is not seen in all patients, but its presence helps in clinching the diagnosis. In this child with worsening pancytopenia despite stopping hydroxyurea and persistence of fever led to suspicion of HLH, which was further confirmed by the presence of high triglycerides and low fibrinogen levels and a bone marrow examination.

Our patient's main diagnostic dilemma was low hemoglobin and cytopenia, which could be either due to chronic hemolysis due to aplastic crisis caused by parvovirus or haemophagocytosis.

Hyperferritinemia could be due to repeated transfusion in sickle beta-thalassemia or secondary to upregulation of heme-oxygenase, a heat shock protein expressed in response to inflammatory cytokines and endotoxin in HLH [4]. Organomegaly is usually a feature of both sickle cell disease (SCD) and HLH. Hypofibrinogemia and haemophagocytosis in bone marrow clinched the diagnosis of HLH in our patient. Retrospectively the response to steroids also attributes to the diagnosis of HLH. HLH is treated in most of the institutes with HLH-2004 protocol. As per the HLH-2004 protocol, HLH is treated for eight weeks with dexamethasone with etoposide and cyclosporine followed by a continuation phase based on clinical response. If there is a presence of central nervous system involvement, intrathecal methotrexate is added. Many reported cases of parvovirus B19 and HLH improved spontaneously or with high dose steroids, as in our case. HLH has been reported previously with sickle cell crisis [5] and in an adult patient with HbH and parvovirus infection [6]. HLH has been rarely reported with other infections, also like Mycobacterium avium, histoplasmosis in patients with sickle cell anemia, and both the reported cases succumbed to their infection [7-8].

## Conclusions

The basis for an association of HLH with sickle cell disease in our patient is HLH triggered by a viral infection. In children with sickle cell disease, persistent fever, and pancytopenia warrant a bone marrow examination to rule out HLH. Secondary HLH is usually associated with infection by EBV, but infection with parvovirus in a sickle beta-thalassemia patient is not commonly reported in children in literature. Early suspicion and prompt treatment were hence utmost necessary to save patients developing this complication.
